# Resistance breakers: a novel approach to tackle biofilm associated infections and antimicrobial resistance

**DOI:** 10.3389/fmicb.2026.1783729

**Published:** 2026-07-20

**Authors:** Zangini Nakazwe, Simone Ries, Sanchita Kar, Mitali Sarkar-Tyson, Aleksandra W. Debowski, Ethan Haese, Keith A. Stubbs, Maud Eijkenboom, K. Swaminathan-Iyer, Andrew Barker

**Affiliations:** 1 The Department of Chemistry, School of Molecular Sciences, The University of Western Australia, Perth, WA, Australia; 2 The Marshall Centre for Infectious Disease Research and Training, School of Biomedical Sciences, The University of Western Australia, Perth, WA, Australia; 3 The Australian Research Council (ARC) Training Centre for Next-Gen Technologies in Biomedical Analysis, School of Molecular Sciences, The University of Western Australia, Perth, WA, Australia; 4 Lixa Ltd., Perth, WA, Australia

**Keywords:** antibiofilm strategies, antimicrobial resistance, antimicrobial synergy, biofilms, combination therapy, resistance breakers

## Abstract

Antimicrobial resistance (AMR) has emerged as one of the greatest global health concerns and its threat to effective treatment of infectious diseases is accelerating. Biofilms remain a major contributor to antimicrobial resistance and persistence of infections. Bacterial biofilms are sessile communities of bacteria surrounded by a self-produced extracellular polymeric substance (EPS). Bacterial cells in biofilms are less susceptible to conventional antibiotics and to host immune effector mechanisms as the cells are protected by the EPS, exhibit altered gene expression as well as heterogeneity in metabolic and physiological states compared to planktonic cells. This poses a challenge for antimicrobial treatments and the development of innovative antibiofilm strategies to combat biofilm associated infections and resistance. This review explores resistance in bacterial biofilms and the recent advancements in resistance breakers: compounds that can inhibit or disrupt mechanisms of resistance to restore clinical efficacy of antibiotics. Different categories of resistance breakers are discussed including matrix disruptors, efflux pump inhibitors, enzyme inhibitors, membrane permeabilizers, quorum sensing inhibitors, and metabolic modulators. Finally, we discuss perspectives on existing translational and clinical challenges.

## 1 Introduction

The problem of AMR in pathogens of clinical importance is on the rise, causing an extensive increase in mortality, morbidity, and substantial economic pressure on healthcare (Pulingam et al., 2022; Antimicrobial Resistance Collaborators, 2022). In 2019, approximately 4.95 million deaths associated with AMR were recorded in 204 countries, and it has been predicted to lead to 39.1 million deaths by 2050 if not mitigated (Pulingam et al., 2022; Antimicrobial Resistance Collaborators, 2022; Inoue, 2019; O'Neill, 2016; Naghavi et al., 2024).

Bacterial pathogens achieve resistance to antibiotics through various mechanisms such as inactivation and modification of antibiotics, a common mechanism in many bacteria which typically involves action by enzymes (Darby et al., 2023). For example, β-lactam antibiotics (penicillins, cephalosporins, and carbapenems) can be hydrolyzed and inactivated by β-lactamases produced by organisms like *Escherichia coli, Pseudomonas aeruginosa*, and *Acinetobacter baumannii* (Darby et al., 2023). Modification of antibiotics can be achieved by the transfer of a chemical group by drug modifying enzymes, thereby rendering antibiotics ineffective with exemplars being acetyltransferases and phosphotransferases that alter aminoglycosides by modifying the hydroxyl or amino groups of the antibiotic, thereby reducing the affinity of the drug to the target (Darby et al., 2023).

Most antibiotics have intracellular targets and need to cross the bacterial cell membrane to exert their activity (Darby et al., 2023). Bacteria have evolved a mechanism to reduce access to the target by reducing membrane permeability. For instance, multidrug resistant *E. coli* isolates were found carrying multiple mutations in a non-specific OmpC channel that changes the charge within the pore, hence, affecting permeability of antimicrobials such as gentamicin and cefotaxime (Darby et al., 2023). Activation of efflux pumps is also known to prevent access to the target (Dhanda et al., 2023). Efflux pumps are transport proteins found in bacterial cell membrane that play a role in transportation of nutrients and extrusion of toxic metabolites and compounds including antibiotics (Pulingam et al., 2022). Antibiotic classes known to be extruded by intrinsic efflux pumps include macrolides, β-lactams, fluoroquinolones, and carbapenems among others (Pulingam et al., 2022). Resistance-nodulation-division efflux pumps [composed of a tripartite AcrAB(Z)-TolC complex] are known to confer the most clinically significant level of resistance in Gram-negative bacteria (Darby et al., 2023).

Target bypass is another known mechanism of resistance in bacteria that involves production of an alternative pathway that bypasses the antibiotic by making the original drug target obsolete (Darby et al., 2023). This can take place through the acquisition of a gene to produce alternative proteins to perform the role of the native protein. Similarly, alteration of the drug target site via mutations in a gene that encodes the target site can lead to antibiotic resistance (Dhanda et al., 2023). For example, fluoroquinolone resistance is achieved as a result of point mutations in genes encoding DNA gyrase and topoisomerase IV enzymes (Nakazwe and Lombe, 2024).

Biofilm formation confers enhanced tolerance to antimicrobial agents and significantly contributes to the global burden of antimicrobial resistance with increases in resistance by up to 1,000-fold (Juszczuk-Kubiak, 2024). Bacterial biofilms are intricate communities of bacteria enclosed in a self-produced matrix known as EPS. Typically, a biofilm consists of 5%−35% microbial cells with EPS making up the remaining volume. The EPS is mainly composed of polymers such as nucleic acids (extracellular DNA), proteins (for example protein subunits from bacterial cell appendages such as flagella and pili), polysaccharides (such as cellulose and glucans), and water (Jamal et al., 2018; Dsouza et al., 2024; Vestby et al., 2020). The biofilm matrix shields the bacterial communities, thereby making them resistant to antimicrobial treatments, tolerant to environmental stresses like nutrient deficiency and pH changes, and conferring the ability to evade host immune responses (Alshaikh et al., 2024). The ability of bacteria to form biofilms is linked to their persistence and antimicrobial treatment failure in human infections (Jamal et al., 2018).

Biofilm-associated infections can be categorized as either device or non-device related and account for 65%−80% of all microbial and chronic infections, respectively (Jamal et al., 2018). In device-related biofilm infections, biofilms usually form on or within medical and non-medical devices such as urinary catheters, peritoneal dialysis catheters, prosthetic devices, contact lenses, mechanical heart valves, ventilators, as well as food processing equipment. Bacterial species that commonly contaminate and produce biofilms on these devices include *E. coli, Enterococcus* spp., *P. aeruginosa, Klebsiella pneumoniae, Streptococcus* spp., *Staphylococcus aureus*, and *Salmonella* spp. among others (Jamal et al., 2018; Alshaikh et al., 2024; Castelijn et al., 2012) (Table 1). In non-device related biofilm infections, biofilms develop on tissues, epithelial linings, wounds, or mucosal surfaces in contact with tissues, thereby causing damage to the affected tissues. For example, periodontitis is an infection of the gums resulting from biofilm formation by different colonizing bacteria due to poor oral hygiene (Del Pozo, 2018). Understanding bacterial mechanisms of resistance as well as physiological and metabolic state changes is imperative in developing unconventional approaches to tackle AMR (Darby et al., 2023; Dhanda et al., 2023; Peng et al., 2025).

**Table 1:** Human biofilm-associated diseases (Dsouza et al., 2024; Donlan and Costerton, 2002).

Infection caused/device colonized	Associated bacteria
Otitis media	*P. aeruginosa, S. aureus, Staphylococcus epidermidis*, and *Haemophilus influenzae*
Prosthetic heart valves	*S. aureus*, Streptococci, and Enterococci
Native valve endocarditis (NVE)	*Streptococci* (including *Viridans Streptococci*), Enterococci, Pneumococci, *and Streptococcus bovis*
Chronic bacterial prostatitis	*E. coli, Klebsiella*, enterobacteria, *Proteus, Serratia, P. aeruginosa*, and *Enterococcus faecalis*
Bacterial lung infections	*S. aureus, Haemophilus influenzae, P. aeruginosa*, and *Burkholderia cepacia*
Periodontitis	*Fusobacterium nucleatum, Peptostreptococcus micros, Eubacterium timidum, Lactobacillus* spp., *Actinomyces naeslundii, Fusobacterium* sp., *Selenomonas sputigena*, and *Porphyromonas gingivalis*
Contact lenses	*S. aureus, P. aeruginosa, E. coli, Proteus* spp., *Serratia* spp., and *S. epidermidis*
Central venous catheters	*P. aeruginosa, S. aureus, K. pneumoniae*, and *E. faecalis*
Urinary catheters	*E. coli, K. pneumoniae, Proteus mirabilis, S. epidermidis, P. aeruginosa, E. faecalis, Providencia stuartii, Enterobacter aerogenes*, and *Acinetobacter calcoaceticus*

## 2 Biofilm development

**Figure 1:**
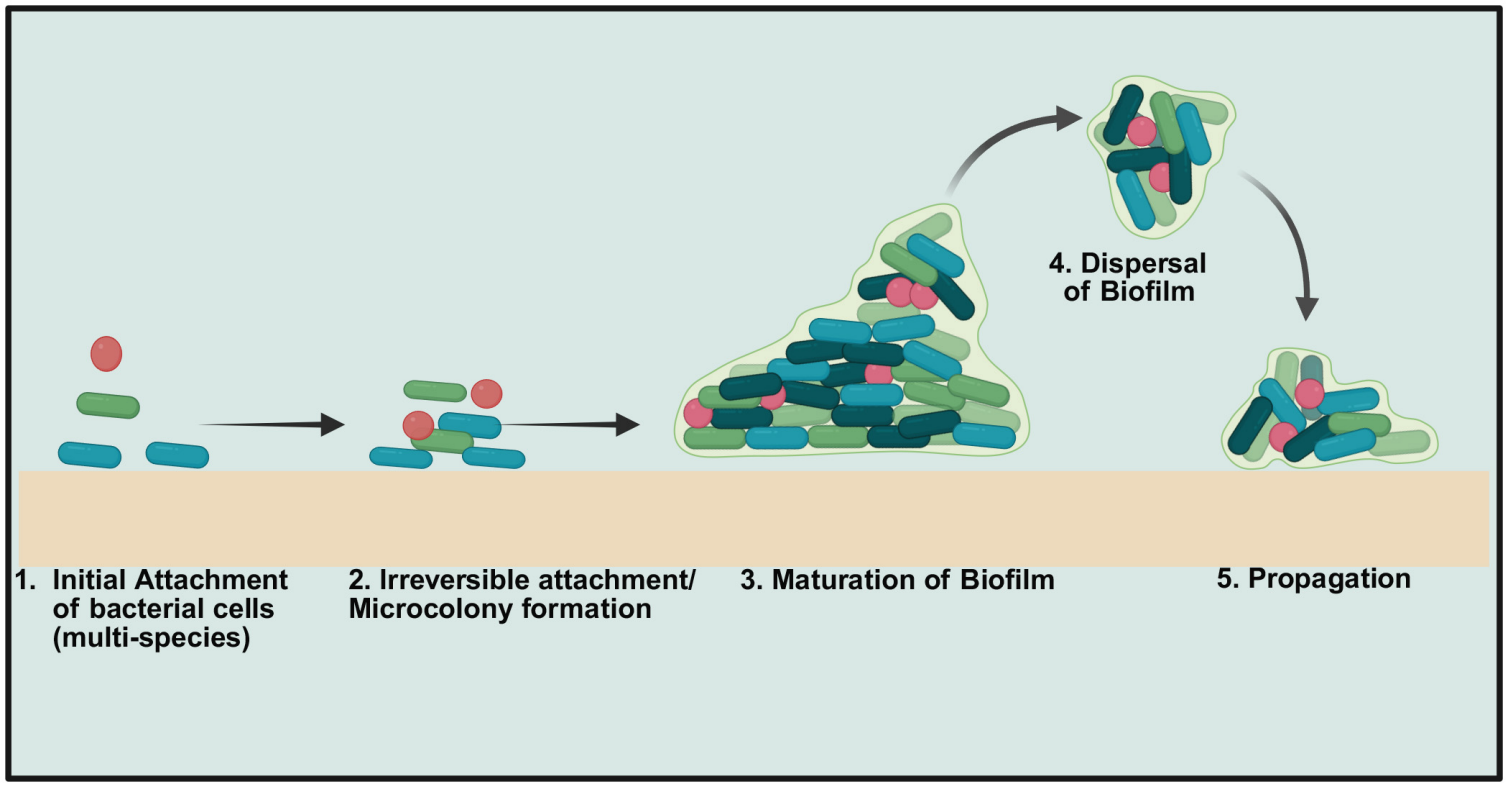
Illustration of a step-by-step biofilm formation process starting with the initial attachment of single cell bacterial cells to a surface, followed by irreversible attachment and development of microcolony, maturation of the biofilm, dispersal of mature biofilm, and propagation of dispersed cells. Figure created with www.biorender.com [Nakazwe, Z. (2026), https://BioRender.com/nbs1enb].

Biofilm formation is a complex process that requires a special type of signaling between microbial cells, known as quorum sensing, which depends on bacterial cell group density and is mediated by signaling molecules known as autoinducers (AIs) (Juszczuk-Kubiak, 2024; Solano et al., 2014). According to genetic studies, biofilm formation requires transcription of different genes compared to planktonic forms. The process of biofilm formation involves several steps, starting with the initial attachment of cells to biotic or abiotic surfaces, irreversible attachment and formation of microcolonies, maturation of the biofilm leading to 3D structures, and finally dispersion of the biofilm (Figure 1) (Jamal et al., 2018; Aparna and Yadav, 2008).

During the initial attachment stage, freely suspended planktonic bacteria adhere to surfaces. The attachment process is facilitated by adhesion proteins, and motility factors like Type IV pili, flagella, and curli fimbriae. Hydrodynamic forces, van der Waals forces, and electrostatic forces play a pivotal role in the reversible attachment of the cells to surfaces with surface features such as roughness and charge impacting adhesion. In stage 2, certain planktonic bacteria undergo a change in orientation to lay flat against the surface. In Gram-negative bacteria, the intracellular signaling molecule bis-(3′-5′)-cyclic dimeric guanosine monophosphate (c-di-GMP) inhibits flagellum-mediated motility and promotes the development of the EPS matrix (Dsouza et al., 2024; Songtanin et al., 2022). This process leads to irreversible attachment and rapid cell proliferation, resulting in the formation of microcolonies. The microcolonies survive by making use of the nutrients available and the conditioning film established. Subsequently, the microcolonies grow and recruit other free-floating bacteria and exogenous debris from the microenvironment in the maturation stage. This process is crucial in the establishment of a distinct mature biofilm 3D structure. The organization of microbes in a mature biofilm result in a “tower” or “mushroom” shape, influenced by nutrient availability, metabolic rate, and aerotolerance (Dsouza et al., 2024). Maturation of the biofilm is followed by a dispersal stage. In this stage, bacteria detach from the mature biofilm due to factors such as fluid movements, limited nutrients, secretory proteins such as proteases dissolving the matrix proteins, and DNases breaking down the extracellular nucleic acids (Dsouza et al., 2024). Microbial cells in this phase upregulate expression of proteins associated with flagella formation, to facilitate bacterial movement to new sites (Jamal et al., 2018). The detached cells relocate and may lodge at other sites to begin a new cycle (Asma et al., 2022).

## 3 Mechanisms of resistance in biofilms

Biofilm associated resistance is multifactorial, comprising a multilayered defense system that includes physical and chemical barriers, as well as genetic, physiological and metabolic components. As a result, conventional antibiotics often fail to effectively reduce bacterial load.

### 3.1 Physical and chemical barrier

The EPS matrix is the physical scaffold of a biofilm that retains bacterial cells in a contained structure and protects the cells from environmental stressors. It acts as a diffusion barrier, delaying or inhibiting penetration of antibiotics to inner layers (Donlan and Costerton, 2002; Luo et al., 2020). Some antibiotics targeting bacterial cells bind to proteins, polysaccharides, or DNA in the matrix or are degraded by enzymes, resulting in decreased concentration and activity of the compounds reaching the target cells (Liu et al., 2024). Within biofilms, chemical microenvironments and gradients of oxygen, nutrients, and pH exist, with these concentration gradients also allowing for drug inactivation. For example, aminoglycosides require oxygen for uptake, but the innermost biofilm layers can be anaerobic. Reduced activity of amikacin in *E. coli* strains was observed under low pH and low oxygen conditions. Under anaerobic conditions, the minimum inhibitory concentration (MIC) of amikacin was 30.0 ± 1.5 μg/mL at pH 7.2 and increased to >50.0 μg/mL at pH 6.0. Meanwhile, under aerobic conditions, the MIC of amikacin was 4.8 ± 0.7 μg/mL at pH 7.2 and 40.0 ± 8.2 μg/mL at pH 6.0 (Bryant et al., 1992). Similarly, biofilms treated with antimicrobials under global anaerobic conditions were more resistant compared to similarly treated aerobic controls (Borriello et al., 2004).

### 3.2 The bacterial cell envelope

A primary challenge in targeting biofilms arises from the complex architecture of bacterial cell envelopes. In Gram-positive bacteria, a thick peptidoglycan layer, intermingled with teichoic acids, offers structural integrity and plays a role in antibiotic interactions (Baek et al., 2018). Gram-negative bacteria on the contrary, possess a dual-layered structure composed of an outer membrane rich in lipopolysaccharides (LPS) and a thin peptidoglycan layer. The outer membrane serves as a selective permeability barrier, aided by porins that restrict the entry of larger molecules while allowing smaller compounds to pass through (Hoque et al., 2016). Overcoming these barriers is critical for improving the efficacy of antimicrobial agents against biofilms.

### 3.3 Altered physiology and metabolic state of bacterial cells with biofilms

The heterogeneity of conditions in biofilms significantly impacts total eradication of bacterial cells by antimicrobial agents. Within a biofilm, bacterial cells are at various stages of the growth cycle with metabolically active cells in the outer layer exposed to nutrients and oxygen, and slow-growing cells as well as metabolically inactive cells in the inner layers (Liu et al., 2024). Many antibiotics, for example β-lactams, and quinolones, target actively growing and dividing cells. In nutrient- and oxygen-limited regions, bacteria have decreased metabolic activity and slow growth (Hall and Mah, 2017). As a result, they enter a stationary or dormant state, reducing susceptibility. Persister cells, a subpopulation that enters a transient dormant state tolerates high antibiotic concentrations without genetic resistance and can repopulate the biofilm after treatment (Luo et al., 2020). Persister cells contribute significantly to the chronic nature of biofilm-associated infections. In addition, it has been demonstrated that the gene expression profile in biofilms is different and diverse compared to planktonic forms, which may promote the development of antimicrobial resistance (Luo et al., 2020).

### 3.4 Genetic adaptation

Biofilm associated cells often show higher efflux activity compared to their planktonic cell counterparts (Luo et al., 2020). Upregulation of efflux pumps actively exports antibiotics out of the cell and can lead to sub-lethal intracellular concentrations of antibiotics, resulting in induced stress responses, enhanced biofilm formation, and accelerated resistance evolution (Liu et al., 2024; Ciofu et al., 2022). Antibiotic resistance genes may be acquired or transferred through horizontal gene transfer (HGT) or spread vertically during replication. High cell density and close contact in biofilms promote plasmid exchange, transposon movement, and acquisition of antibiotic resistance genes. Extracellular DNA (eDNA) from lysed cells in the matrix serves as a reservoir of antibiotic resistance genes.

### 3.5 Quorum sensing mechanisms

Bacterial cells in biofilms are compactly arranged, allowing for mechanisms such as quorum sensing, which is an intercellular communication system used by bacteria to coordinate community behaviors in response to cell population densities using chemical signal molecules [acyl-homoserine lactones (AHLs), frequently used in Gram-negative bacteria, and autoinducing peptides (AIPs) in Gram-positive bacteria]. When the threshold concentration of signal molecules is reached, it is detected by receptors. This triggers changes in gene expression leading to synchronized behaviors across the community, and results in social activities such as biofilm formation (Whiteley et al., 2017). In biofilms, some species produce protective enzymes such as catalases, β-lactamases, or aminoglycoside-modifying enzymes that inactivate antibiotics locally. These enzymes can protect neighboring cells in the biofilm. Additionally, studies have shown that quorum sensing in biofilms can also upregulate efflux pumps, stress responses, and virulence factors (Alcalde-Rico et al., 2016).

## 4 Strategies for resistance breaking

**Figure 2:**
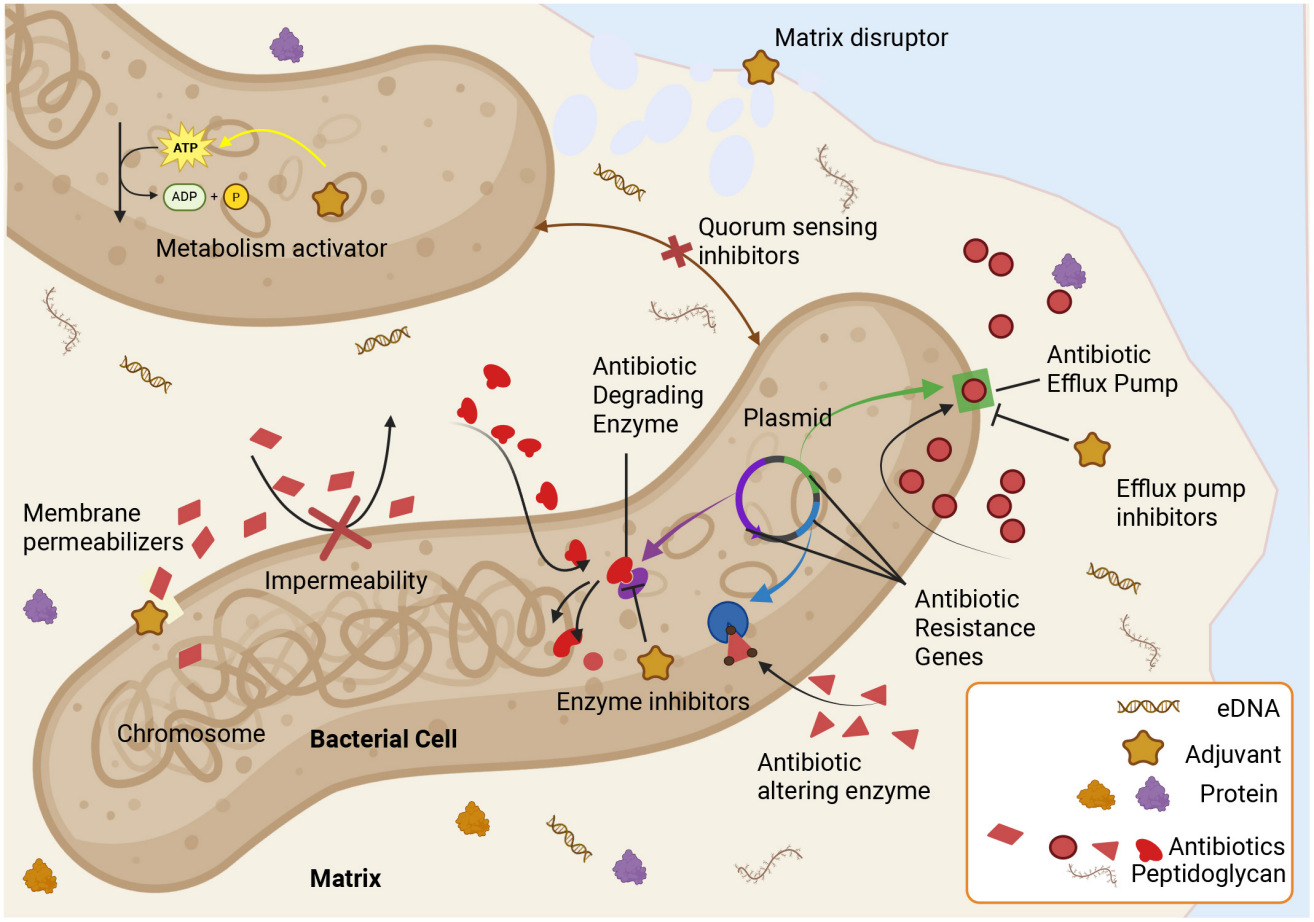
Mechanisms of resistance breaking. Figure generated with www.biorender.com [Ries, S. (2026), https://BioRender.com/esw8ogw].

For many years, conventional antibiotics have been used to treat biofilm associated infections. However, studies have shown a lack of effectiveness of conventional antibiotics in treating biofilms compared to planktonic cell counterparts (Olson et al., 2002). Hence, the development of strategies to inhibit biofilm formation has emerged as an area of interest. Resistance breaking is one of the promising strategies to combat antimicrobial resistance. The strategy focuses on repurposing non-antibiotic drugs to counteract resistance mechanisms, optimizing treatment effectiveness, and promoting the development of novel therapeutic agents to combat resistance. Antibiotic resistance breakers are compounds that potentially target and reverse underlying bacterial resistance mechanisms to restore the clinical efficacy of traditional antibiotics (Laws et al., 2019). Resistance breakers have been classified as direct, indirect, narrow, universal, or host-modulating, depending on their mode of action. Synergistic combinations, particularly those involving resistance breakers, are suggested to work by targeting the multilayered defense system discussed above. The primary classes discussed here include efflux pump inhibitors, enzyme inhibitors, membrane permeabilizers, metabolic modulators, and matrix and signaling disruptors (Figure 2).

### 4.1 Matrix disruptors and biofilm dispersal

Biofilm dispersal represents a promising strategy to increase bacterial susceptibility to antibiotics. Biofilms are primarily composed of an EPS matrix, which includes exopolysaccharides, extracellular DNA, and extracellular proteins. These components are potential targets for preventing biofilm formation or disruption. Various approaches have been employed to achieve biofilm eradication, including enzymatic degradation, antimicrobial peptides, nanoparticles, and electrochemical methods, each targeting different aspects of the EPS matrix to compromise biofilm integrity.

Several enzymes in the glycoside hydrolase family: Dispersin B (DspB), PgaB, alginate lyase, PsIG, PelA, amylase, and cellulase act as biofilm-dispersing agents by hydrolysing the polysaccharide components of the EPS matrix (Wang et al., 2023). DspB is a widely studied enzyme responsible for biofilm degradation in various bacterial strains: *S. aureus, Aggregatibacter actinomycetemcomitans, S. epidermidis, A. baumannii, K. pneumoniae, E. coli, Burkholderi*a spp., *Actinobacillus pleuropneumoniae, Y. pestis*, and *P. fluorescens* (Wang et al., 2023). Treatment with 40 μg/mL of DspB for 30 mins at 30 °C has been shown to effectively degrade the biofilm of *A. pleuropneumoniae* (Kaplan et al., 2004). In an *in vitro* assay, DspB was shown to be highly effective in dispersing biofilms of *E. coli, P. fluorescens, Y. pestis* and *A. pleuropneumoniae* at a concentration of 10–50 μg/mL (Itoh et al., 2005). In an *in vivo* assay, DspB (200 μg/mL) significantly reduced methicillin-resistant *S. aureus* (MRSA) infection and promoted wound healing in chronic wound model (Gawande et al., 2014). In an *in vitro* assay, it also inhibited 10%−40% biofilm formation of two *S. aureus* strains (Izano et al., 2008). Recombinant DspB has been also shown to effectively break down established biofilms of *S. epidermidis* at 40 μg/mL, particularly when followed by treatment with 1 mg/mL protease (proteinase K or trypsin) (Chaignon et al., 2007). However, some pathogens, such as *B. cenocepacia* and *Achromobacter xylosoxidans*, form biofilms that are resistant to treatment with recombinant DspB, highlighting variability in its biofilm disrupting efficacy across bacterial species (Dobrynina et al., 2015). PgaB, another glycoside hydrolase, disrupted biofilms formed by different pathogens: *Bordetella pertussi*s, *S. carnosus, S. epidermidis*, and *E. coli* within 2 h at a concentration of 2 μM (Little et al., 2018). A recent study demonstrated that 250 U/mL of purified marine alginate lyase (AlyP1400) can degrade *P. aeruginosa* biofilms and enhance the effectiveness of the antibiotic tobramycin (Daboor et al., 2021).

Enzymatic degradation of extracellular proteins in EPS matrix is another effective way to eradicate biofilms. Diverse proteinases: proteinase K, trypsin, pepsin, aureolysin, and peptidase M16 among others have been studied as biofilm-disrupting agents (Wang et al., 2023). Proteinase K, for example, demonstrated *S. aureus* antibiofilm activity at 2 μg/mL. It inhibited biofilm formation by interfering with early adhesion, as well as dispersing established biofilms at 24 and 48 h. Its efficiency increased in a dose-dependent manner, with approximately 36% reduction observed at 2 μg/mL and up to ~76% disruption at the highest tested concentration of 250 μg/mL (Kumar Shukla and Rao, 2013). In addition, it enhanced antibiotic efficacy with reductions in MIC corresponding to a 3-log decrease when combined with gentamicin or streptomycin, and a 1.3-log decrease with ampicillin. This synergistic effect was also observed against other pathogens, where proteinase K combined with antibiotics effectively degraded preformed biofilms of *S. aureus, E. coli, S. lugdunensis, S. haemolyticus, Listeria monocytogenes, Gardnerella vaginalis*, and *Bdellovibrio bacteriovorus* (Nguyen and Burrows, 2014; Cui et al., 2016; Radwan et al., 2022; Hu et al., 2020). A combination of 50 μg/mL proteinase K and 0.5 mg/mL thyme oil resulted in a 3.84 log reduction in *E. coli O157:H7* biofilms after 48 h (Cui et al., 2016). In an *in vitro* assay, proteinase K completely inhibited *L. monocytogenes* biofilm formation at 0.8 μg/mL and fully eradicated established biofilms within 1 h at concentrations ≥1.6 μg/mL (Nguyen and Burrows, 2014). Trypsin alone was able to reduce the biomass of preformed 24-h-old biofilms of both *P. aeruginosa* and *Enterococcus faecalis*. However, this enzyme could not completely remove biofilms regardless of concentration and treatment duration. Interestingly, when combined with pepsin at 1 mg/mL, trypsin reduced the MIC of carvacrol (a natural monoterpenoid phenol) to half of its original value. This combination also detached 40%-50% of the *P. aeruginosa* biofilms as well as the *E. faecalis* biofilms (Mechmechani et al., 2023).

Extracellular DNA, another key component of the EPS matrix, represents an alternative target for biofilm eradication. Several DNases have been investigated for this purpose, including DNase I, Nuclease Xds, Nuclease Dns (VcEndA), Streptodornase (Varidase), and NucB (Wang et al., 2023). DNase I, a widely used pancreatic endonuclease, demonstrated greater efficacy against early-stage biofilms, with substantial dissolution observed in biofilms up to 60 h old. In contrast, biofilms older than 84 h exhibited significant resistance due to structural reinforcement by exopolysaccharides and proteins, as well as the production of proteolytic enzymes that can digest DNase I locally (Whitchurch et al., 2002). Furthermore, structural changes in eDNA from B-form to DNase-resistant stable Z-form with distinct nucleotide geometry confer structural integrity to the biofilm matrix (Buzzo et al., 2021). Recombinant human DNase I has been clinically employed in cystic fibrosis patients to reduce the viscosity of DNA-rich sputum and improve pulmonary function (Wagener and Kupfer, 2012). Streptodornase was also shown to be very effective in disrupting *P. aeruginosa* biofilms *in vitro* at >625 U/mL ($\frac{1}{4}$ clinical dose) after 3-hour treatments at 37 °C, highlighting its potential against focal infections such as UTIs. The same result was also observed for DNase I. NucB, a DNase from *Bacillus licheniformis*, effectively degraded preformed biofilms of staphylococci and streptococci from chronic rhinosinusitis infections at 3 μg/mL within 1 h, highlighting its therapeutic potential (Shields et al., 2013). At the same concentration, NucB has also been shown to disperse a variety of mono- and mixed-species bacterial biofilms (Shakir et al., 2012).

Bacterial DNA-binding proteins, particularly DNABII, play a crucial role in transition of eDNA from B- to more stable Z-form in mature biofilms (Buzzo et al., 2021). Disruption of DNABII proteins destabilizes the eDNA structure, leading to rapid loss of biofilm integrity (Goodman et al., 2011). Targeting the DNABII proteins with an anti-DNABII antibody has therefore, emerged as a promising antibiofilm strategy. CMTX-101, an anti-DNABII monoclonal antibody disrupted biofilms formed by MRSA and tobramycin-resistant *P. aeruginosa* (Rogers et al., 2022). Similarly, murine monoclonal antibodies directed against DNABII proteins (conserved DNA binding proteins HU and IHF) disrupted mixed-species biofilms wherein non-typeable *H. influenzae* co-partnered with other respiratory tract pathogens (Jurcisek et al., 2022). A phase 1b/2a clinical trial supported by the Cystic Fibrosis Foundation is currently evaluating CMTX-101 safety in combination with inhaled antibiotics in cystic fibrosis patients (ClinicalTrials.gov Identifier: NCT06159725). The Phase 1b portion was completed in October 2024 with no major safety signals, and the Phase 2a portion is ongoing, with interim results expected in 2025.

Bacteriophages (phages), viruses that only infect specific bacterial strains have emerged as promising antimicrobial agents in response to the global rise of antimicrobial resistance (Grooters et al., 2024; Pattnaik et al., 2024). Their high host specificity, self-amplifying nature and ability to target multidrug-resistant pathogens make them attractive alternatives or adjuvants to conventional antibiotics (Mobarezi et al., 2025; Dicks and Vermeulen, 2024). Increasing evidence has highlighted the importance of phages in combating bacterial biofilms through their capacity to degrade the biofilm matrix and promote biofilm disruption (Mobarezi et al., 2025; Liu et al., 2022). Phages produce depolymerases among other enzymes that breakdown biofilm structures (Harper et al., 2014). Additionally, phages penetrate biofilm structures through water channels. Coulter et al. utilized species-specific phages in combination with tobramycin to treat *E. coli* and *P. aeruginosa* biofilms: phage T4 was used against *E. coli* strain B, and phage PB-1 was used against *P. aeruginosa* PAO1. The study demonstrated a significant reduction in antibiotic-resistant and phage-resistant cells in both biofilm systems (Coulter et al., 2014). In a study by Szymczak et al., T7 phages armed with silver nanoparticles exhibited enhanced antibiofilm activity against *E. coli* biofilms compared with phage treatment alone. The phage-silver nanoparticle conjugates effectively penetrated and disrupted mature biofilms. These findings highlight the potential of combining bacteriophages with complementary antimicrobial technologies to overcome inherent tolerance of biofilm-associated bacteria (Szymczak et al., 2024). Building upon this concept, nanoparticles themselves have attracted considerable interest as promising agents for the disruption of microbial biofilms. Nitric oxide (NO)-releasing silica nanoparticles (8 mg/mL) eradicated ≥99% of biofilm cells of *P. aeruginosa, E. coli, S. aureus, S. epidermidis*, and *C. albicans with* lower cytotoxicity toward fibroblasts (the key cells in connective tissue repair) than clinical antiseptics, thereby supporting their wound healing benefits (Hetrick et al., 2009). Positively charged chitosan nanoparticles loaded with oxacillin and DNase I (CSNP-DNase-Oxa) were tested against clinical *S. aureus* biofilms, with the lowest concentration of oxacillin (0.0625 μg/mL) in the complex detaching 70% of 24-h biofilms after 2 h of treatment, and the system showed no cytotoxicity in HaCaT cell line (human immortalized keratinocytes) (Tan et al., 2018).

#### 4.1.1 Challenges and future directions

Despite extensive research into biofilm-dispersing agents, several drawbacks remain. Firstly, most studies have been conducted *in vitro* on mono-species biofilms, limiting extrapolation to the complex, multispecies biofilms found *in vivo*. The lack of reliable biofilm models that mimic natural microbial colonization further constrains progress. Secondly, the presence of antibiotics can negatively influence enzyme stability and activity when co-administered. This occurs because antibiotics may directly interact with enzymes, alter the local microenvironment, or induce bacterial stress responses that affect enzyme function. For example, sub-MIC concentrations of erythromycin or doxycycline can modulate *S. aureus* micrococcal nuclease activity (Rosman et al., 2021). In addition, antibiotics with varied pKa values may alter enzyme function if co-administered, particularly if mixed in the same infusion bag, where pH-mediated inactivation may occur before *in vivo* delivery (Thapa et al., 2019). Therefore, enzymes with broad pH tolerance, thermostability, durability in organic solvents, and resistance to host-related inhibitory interactions are preferred. Despite these limitations, stable enzymes such as peptidase M16 and proteinase K demonstrate promising therapeutic potential (Ebeling et al., 1974; Saggu et al., 2019). Thirdly, the dispersal of microbes presents its own challenges. While it can improve antimicrobial efficacy and immune access, dispersal may also increase the risk of sepsis or enhanced virulence (Connolly et al., 2011), particularly in fungal biofilms, where it can exacerbate infection spread. Finally, while glycosidases and other biofilm-degrading enzymes hold promise for disrupting microbial biofilms, their potential cytotoxicity presents a significant challenge. In one study, 12 glycosidases, including alginate lyase, amylase, amyloglucosidase, xylanase, cellulase, and pectinase, were found to adversely affect human epithelial fibroblasts and normal colonic cells (Redman et al., 2021). Given the extensive data available, researchers and industry should focus on translating these findings to patient care through *in vivo* assays, clinical trials, and evaluation of toxicity and stability using complex biofilm models.

### 4.2 Efflux pump inhibitors

Efflux pumps are one of the major contributors to increased resistance against many kinds of antimicrobial chemicals, such as antibiotics, antiseptics, and detergents, in bacteria. In addition to conferring resistance, efflux pumps also play roles in bacterial physiology, and many are crucial for bacterial pathogens to cause infection and to form biofilms (Fuji et al., 2025). To date, five different types of efflux pumps have been reported: resistance-nodulation-division (RND), major facilitator superfamily (MFS), ATP-binding cassette (ABC), multidrug and toxic compound extrusion (MATE), and small multidrug resistance (SMR) (Ahmed et al., 2019). Among these, RND type pumps are well-studied and most important because of their tripartite complex consisting of an inner membrane protein, a periplasmic protein, and an outer membrane protein (Almeida et al., 2024). The inner membrane protein possesses a multi-site binding pocket and can recognize various chemicals as its substrate. These efflux types are more efficient as the drug is released in the periplasm and RND pumps capture the drug in this space (Nikaido and Takatsuka, 2009). AcrAB, OqxAB, EefAB, KexD, AcrAB-TolC, AcrD, AcrEF, MdtABC, and MexAB-like pumps are RND type pumps that have been reported in *K. pneumoniae* (Almeida et al., 2024), *E. coli* (Almeida et al., 2024), and *Enterobacte*r spp. (Guérin et al., 2016; Venter et al., 2015).

Efflux pump inhibition represents a promising strategy to mitigate AMR and disrupt biofilm development. Efflux pump inhibitors (EPIs) interfere with bacterial transport systems responsible for expelling antibiotics. By blocking drug extrusion, EPIs promote the intracellular accumulation of antibiotics, thereby reducing efflux-mediated resistance. Efflux pump activity can be suppressed through several approaches: (i) suppressing efflux pump gene expression by interfering with their regulatory mechanisms, (ii) modifying antibiotics so they are no longer recognized as pump substrates, (iii) preventing the proper assembly of functional efflux pumps during the translation process, (iv) blocking the active site to hinder substrate binding, and (v) disrupting the energy source that drives pump activity (Ashima and Bhardwaj, 2012).

EPIs can be categorized according to their origin and functional mechanisms. Correspondingly, EPIs are derived from diverse sources, including natural products, synthetic compounds, and microbial metabolites. Regarding their mode of action, EPIs are broadly classified into two categories: inhibitors that disrupt the energy supply required for efflux activity, and inhibitors that compete with antibiotics to bind directly to the efflux pump protein. Several EPIs have been investigated to date (Sharma et al., 2019). Among the most extensively studied EPIs are carbonyl cyanide *m*-chlorophenylhydrazone (CCCP), 1-(1-naphthylmethyl)-piperazine (NMP), and phenylalanine-arginine beta-naphthylamide (PAβN; Table 2).

**Table 2:** Representative chemical structures of efflux pump inhibitors (EPIs).

Compound	Chemical structure	Mechanism/target	Antibiotic effect	Organism	References
Carbonyl cyanide *m*-chlorophenylhydrazone (CCCP)	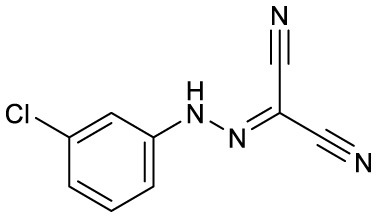	Disruption of proton motive force	Restore the activity of tetracycline; inhibit biofilm formation	*Helicobacter pylori* and *Klebsiella* spp., *A. pleuropneumoniae, Salmonella, E. coli, K. pneumoniae, P. aeruginosa*, and *S. aureus*	Nikaido and Takatsuka, 2009; Guérin et al., 2016; Anoushiravani et al., 2009; Fenosa et al., 2009; Osei Sekyere and Amoako, 2017; Gallo et al., 2003; Malléa et al., 2003
		Effect on bacterial metabolic process	Synergistic effect with carbapenem	Enterobacteriaceae	Venter et al., 2015
1-(1-naphthylmethyl)-piperazine (NMP)	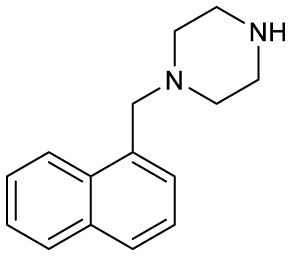	Competitive binding to efflux pump	Enhance chloramphenicol activity	*E. aerogenes, P. aeruginosa*	Ahmed et al., 2019; Malléa et al., 2003
			Reduce virulence; Inhibit biofilm formation	*P. aeruginosa and E. coli, A. pleuropneumoniae, Salmonella, E. coli, K. pneumoniae, P. aeruginosa*, and *S. aureus*	Chevalier et al., 2004; Renau et al., 1999; Anoushiravani et al., 2009; Fenosa et al., 2009; Osei Sekyere and Amoako, 2017; Gallo et al., 2003; Malléa et al., 2003
Alkoxyquinoline derivative (EPI)	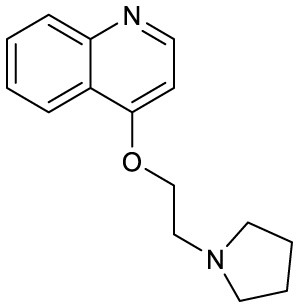	Competitive binding to efflux pump	Enhance chloramphenicol and ciprofloxacin activity	*E. aerogenes*	Ashima and Bhardwaj, 2012; Sharma et al., 2019
Phenylalanine-arginine-β-naphthylamide (PaβN)	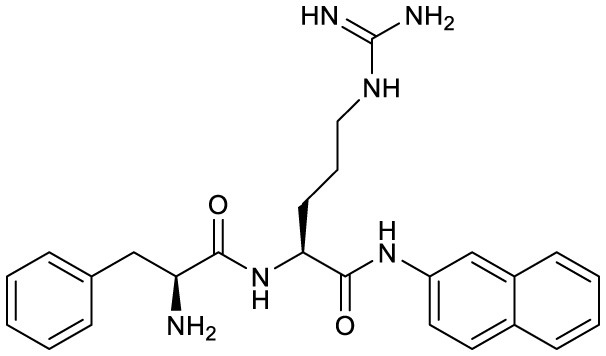	Competitive binding to efflux pump	Enhance chloramphenicol and ciprofloxacin activity	*E. aerogenes, A. pleuropneumoniae, Salmonella, E. coli, K. pneumoniae, P. aeruginosa*, and *S. aureus*	Ahmed et al., 2019; Anoushiravani et al., 2009; Fenosa et al., 2009; Osei Sekyere and Amoako, 2017; Gallo et al., 2003; Malléa et al., 2003
		Modulate gene expression	Reduced virulence	*P. aeruginosa and E. coli*	Chevalier et al., 2004; Renau et al., 1999
Thioridazine	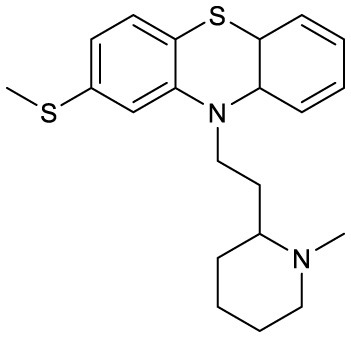	Disruption of proton motive force	Intracellular antibiotic concentration increases	*S. aureus* and *P. putida*	Li et al., 2016

#### 4.2.1 Proof of concept and clinical promise

CCCP is an ionophore that disrupts the proton motive force, thereby inhibiting RND-type efflux pumps. CCCP not only inhibits efflux pumps but also influences bacterial metabolic processes. PAβN and NMP are broad-spectrum RND efflux pump inhibitors that function by competing with antibiotics and binding directly to efflux pumps such as the AcrAB-TolC efflux system in *Enterobacteriaceae* (Chevalier et al., 2004) and MexAB-OprM efflux system in *P. aeruginosa* (Renau et al., 1999). CCCP has been reported to restore the activity of tetracycline in *Helicobacter pylori* and *Klebsiella* spp. (Anoushiravani et al., 2009; Fenosa et al., 2009). It also shows synergistic effects with carbapenems, which seem to be independent of efflux pump inhibition, possibly due to induction of a metabolically inactive state in bacterial cells (Osei Sekyere and Amoako, 2017). An alkoxyquinoline derivative EPI (quinoline based compounds) increased antibiotic accumulation in *E. aerogenes* cells when incubated with chloramphenicol and increased the strain sensitivity to chloramphenicol approximately 20-fold at 2 mM (Gallo et al., 2003) and 16-fold at 0.2 mM (Malléa et al., 2003). Similar results were also observed in the presence of 0.2 mM PAβN (Chevalier et al., 2004). Another study demonstrated that 27 μM PAβN modulated gene expression in *P. aeruginosa* and *E. coli*, leading to reduced virulence and strong efflux pump inhibition (Rampioni et al., 2017; Misra et al., 2015). Both PAβN and CCCP have demonstrated significant inhibitory effects on biofilm formation across multiple species, including *A. pleuropneumoniae* (Li et al., 2016), *Salmonella* (Baugh et al., 2012), *E. coli* (Kvist et al., 2008; Baugh et al., 2014), *K. pneumoniae* (Kvist et al., 2008), *P. aeruginosa* (Baugh et al., 2014; Liu et al., 2010), and *S. aureus* (Kvist et al., 2008; Baugh et al., 2014). CCCP inhibited *Salmonella* biofilm formation at 1 μg/mL, while PAβN required 32 μg/L to achieve a similar effect. Another study demonstrated that a thioridazine/PAβN combination inhibited *E. coli* and *K. pneumoniae* biofilm formation up to 99% at 50 μg/mL each. A similar reduction was also observed using NMP. Thioridazine and PAβN (20 μg/mL each) decreased biofilm formation by 50–70% in *S. aureus* and 40%−50% in *P. putida* after 24 h, while NMP showed no significant activity (Kvist et al., 2008). Under dynamic flow cell conditions, thioridazine in combination with NMP (50 and 100 μg/mL) demonstrated substantial antibiofilm activity, reducing *E. coli* biofilms by 83% after 24 h (Kvist et al., 2008). Importantly, the effectiveness of biofilm inhibition was strongly strain dependent. More recently, PAβN (8 μg/mL) inhibited biofilm formation in carbapenem-resistant *P. aeruginosa* (Li et al., 2022). When PAβN (50 μg/mL) combined with iron chelators, it significantly reduced *P. aeruginosa* biofilm formation. At the same concentration, PAβN alone or in combination with iron chelators also lowered the ciprofloxacin MIC from 0.2 to 0.05 μg/mL (Liu et al., 2010).

Nanoparticles have also been shown to inhibit efflux pumps and function as antibiofilm agents. For example, copper nanoparticles (CuNPs) were effective in suppressing efflux activity in wild-type *S. aureus* and *P. aeruginosa* at 0.5 × MIC (0.065 mM) and at 1 × MIC (0.13 mM) concentration; they significantly inhibited single-species biofilms of *S. aureus* and *P. aeruginosa* (Christena et al., 2015).

#### 4.2.2 Challenges and future directions

Although EPIs hold considerable therapeutic potential, their toxicity to human cells remains a major barrier to their clinical translation (Li et al., 2015). To address this limitation, studies have investigated combination strategies involving EPIs and membrane-permeabilizing antimicrobial peptides (AMPs), which can enhance antibiotic uptake at lower concentrations. For example, polymyxin B non-apeptide (PMBN)—a papain-digested derivative of polymyxin B with reduced toxicity in a murine model has been shown to restore antibiotic activity. When a PMBN/PAβN combination was used at 1 μg/mL, the MICs of azithromycin and doxycycline decreased by 512 and 128-fold, respectively (from 128 to 0.25 μg/mL and from 64 to 0.5 μg/mL). Similar synergistic effects were also observed with other antibiotics, including levofloxacin, ceftazidime, piperacillin, and aztreonam, as well as when NMP was employed as an alternative to PAβN (Ferrer-Espada et al., 2019). Recently, metal nanoparticles have shown potential as EPIs by reducing the MICs of antibiotics in *E. coli, K. pneumoniae, A. baumannii*, and *Enterobacter* spp. However, this approach remains in its infancy, requiring further biochemical and clinical validation.

Other considerable challenges in the clinical translation of EPIs are economic constraints, structural limitations of natural compounds (complex and bulky structure, making synthesis difficult) and synthetic molecules (poor solubility, limited cell permeability, and toxicity), as well as incompatibilities between EPIs and antibiotics (Gandhi et al., 2013). Additionally, the complexity and diversity of bacterial resistance mechanisms, along with the narrow substrate specificity of EPIs (Lomovskaya et al., 2001) and limited pre-clinical and clinical evidence, raise concerns about their broad applicability. Overcoming these obstacles will be critical to fully harness the potential of EPIs as adjuncts in combating antimicrobial resistance.

Due to all these challenges, there is minimal industry involvement in the development of new EPIs for pharmaceutical use. Nonetheless, some companies have already come forward. TAXIS Pharmaceuticals (New Jersey, USA) has recently received funding from CARB-X to advance the development of EPIs targeting multidrug-resistant *P. aeruginosa* (NORTH BRUNSWICK, 2024).

### 4.3 Enzyme inhibitors

Enzymes play significant roles in various biological processes, and their inhibition has emerged as a viable strategy to enhance the efficacy of existing antibiotics. A widespread mechanism of resistance exhibited by most pathogenic bacteria involves production of enzymes that inactivate antibiotics via degradation or modification of either the antibiotic or its target by the transfer of a chemical group (Darby et al., 2023). Enzymatic degradation alters the chemical structure of the antibiotic rendering it unable to bind its target, while enzymatic modification involves the addition of chemical groups that similarly reduce drug efficacy; both mechanisms constitute major contributors to clinical resistance (Darby et al., 2023). For example, β-lactam antibiotics are a widely used and effective class of antibiotics used to treat bacterial infections, but their efficacy is reduced through hydrolysis by β-lactamases. β-Lactams target the penicillin binding protein (PBP): a key enzyme required for peptidoglycan biosynthesis. Bacteria gain resistance to β-lactams through the production of β-lactamases, which cleave the β-lactam ring, consequently making the antibiotic ineffective (Venter, 2019). The transfer of a chemical group by drug modifying enzymes also renders antibiotics ineffective. For example, aminoglycosides can be modified by acetyltransferases, nucleotidyltransferases or phosphotransferases, which act to modify amino or hydroxyl groups in the antibiotic. High-level aminoglycoside resistance is mediated by methyltransferase-driven modification of the16S rRNA, blocking drug interaction with the 30S ribosomal subunit (Srinivas et al., 2023). Several other enzymes have been discovered that can modify the various classes of antibiotics, including macrolides, rifamycins, phenicols, streptogramins, and lincosamides (Darby et al., 2023). Recent studies have revealed new enzymes as potential drug targets. For instance, targeting the β-ketoacyl-acyl carrier protein synthase III (FabH) has drawn attention due to its selective activity in bacterial cell wall synthesis, which is fundamental for bacterial survival and pathogenicity (Verma et al., 2025). This approach represents a shift from conventional targets, potentially reducing resistance development.

#### 4.3.1 New developments

The development of enzyme inhibitors as adjunct therapies is a proven strategy that aims to restore the efficacy of existing antibiotics. For instance, vaborbactam is a novel, non-β-lactam that was developed to inhibit serine carbapenemases, specifically *K. pneumoniae* carbapenemases (KPC β-lactamases) (Hecker et al., 2015). In past studies, a combination of vaborbactam and meropenem exhibited a strong inhibitory effect against carbapenem resistant Gram-negative bacteria (Hackel et al., 2018; Lomovskaya et al., 2017). Recent advances in β-lactamase inhibitor development have led to the introduction of diazabicyclooctane-based agents, including avibactam, durlobactam, and relebactam, alongside boronic acid inhibitors such as vaborbactam and taniborbactam, which is currently undergoing phase III clinical evaluation (Darby et al., 2023; Hecker et al., 2015) (Table 3). Most recently, the FDA approved the use of vabomere (a meropenem—vaborbactam combination) for complicated urinary tract infections caused by resistant Gram-negative organisms, and imipenem—relebactam also shows viable clinical potential, confirming the great promise of this method of resistance reversal (Darby et al., 2023; Venter, 2019). Many other β-lactam compounds, including sulbactam (combined with ampicillin), tazobactam (with piperacillin), and clavulanic acid (with amoxicillin), are clinically important β-lactamase inhibitors (Laws et al., 2019; Hecker et al., 2015). Furthermore, sulfonamide derivatives have also shown potential in inhibiting bacterial carbonic anhydrases, facilitating the treatment of infections caused by resistant strains (Delp et al., 2024). These inhibitors can improve the efficacy of antibiotics by preventing bacteria from neutralizing the drugs, thereby overcoming resistance mechanisms (Venter, 2019; Hecker et al., 2015). Pyrrole-2-carboxylates (PyCs)have recently been developed as novel metallo-β-lactamase inhibitors that potentiate carbapenem activity against resistant Gram-negative bacteria. Unlike conventional zinc-chelating inhibitors, optimized analogs have demonstrated potent inhibition of di-Zn(II) ion containing metallo-β-lactamases (New Delhi metallo-β-lactamase-1, Imipenemase-1, Verona integron encoded MBL-1, and Verona integron encoded MBL-2) by trapping the catalytically essential di-Zn(II) ion bridging hydroxide within the enzyme active site, thereby preventing β-lactam hydrolysis (Singha et al., 2026).

**Table 3:** Representative chemical structures of enzyme inhibitors (Darby et al., 2023; Dhanda et al., 2023; Lomovskaya et al., 2001).

Compound	Chemical structure	Target enzyme	Mode of action	Organism
Vaborbactam	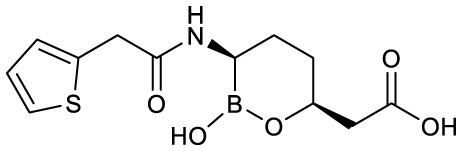	Class A (especially KPC)	Boronic acid inhibitor	KPC-producing Enterobacterales
Avibactam	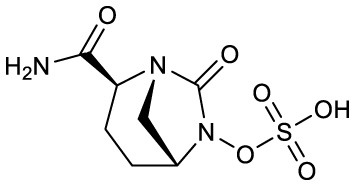	Class A (ESBL, KPC), Class C (AmpC), and some class D (OXA-48)	Reversible covalent inhibitor	KPC producers, AmpC, Enterobacterales, OXA-48
Durlobactam	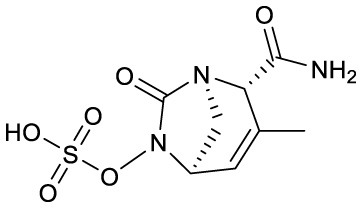	Class A, C, D (*Acinetobacter* OXA enzymes)	Diazobicyclooctane inhibition	Carbapenem-resistant *A. baumannii*
Relebactam	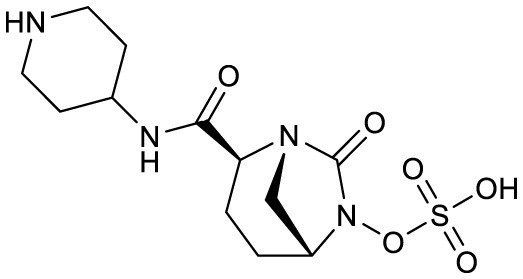	Class A (KPC), Class C (AmpC)	Reversible inhibitor	KPC, AmpC-producing organisms
Sulbactam	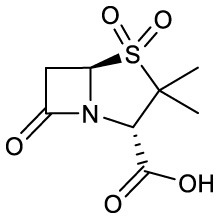	Class A non-carbapenemases (GES, CTX-M, SHV, and TEM), Class D non-carbapenemases	Irreversible inhibitor	*Acinetobacter* spp., ESBL producers
Tazobactam	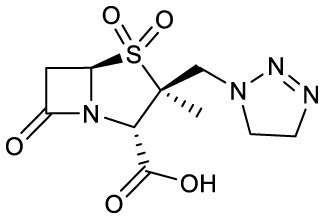	Class A (some ESBL: CTX-M, SHV, and TEM), Class D non-carbapenemases	Irreversible inhibitor	ESBL *E. coli, Klebsiella*
Clavulanic acid	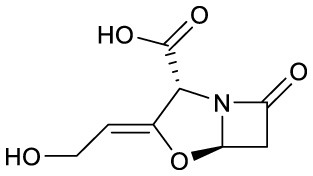	Class A (ESBL-CTX-M, TEM, and SHV), Class D non-carbapenemases	Irreversible enzyme inactivation	ESBL-producing *E. coli, Klebsiella*

#### 4.3.2 Challenges and outlook

Although the use of enzyme inhibitors holds high potential, challenges remain. Enzyme inhibitors are less effective against diverse multiple resistance mechanisms such as changes in metabolic processing of the drug, mutations in target enzymes, and activation of alternative signaling pathways (Darby et al., 2023; Dhanda et al., 2023). In addition, enzyme inhibitors require susceptibility diagnostics before deployment, restricting the clinical flexibility (Dingle and Pitout, 2022; Lawrence et al., 2024). The interplay between enzyme inhibitors and drug resistance is multifaceted, driven by diverse biological mechanisms that undermine existing therapeutic approaches. The development of effective inhibitors often calls for extensive understanding of resistance mechanisms and enzymatic pathways, along with thorough assessment for safety and efficacy (Del Prete and Pagano, 2024). Future research should focus on optimizing existing compounds, understanding the underlying mechanisms of resistance, and discovering novel pathogen-agnostic inhibitors that can combat multiple resistance mechanisms.

### 4.4 Membrane permeabilizers

Given the barriers posed by the bacterial cell envelope, membrane permeabilizers have emerged as a promising approach to enhance the efficacy of conventional antibiotics against biofilm-associated infections. These compounds disrupt bacterial cell membranes, causing irreparable damage while enabling antimicrobial agents to act from within. This approach is particularly effective for Gram-negative bacteria, where an intact outer membrane represents a substantial barrier to many antibiotics (Du et al., 2023). Research indicates that combining permeabilizers with traditional antibiotics can significantly improve their antibacterial efficacy, especially in biofilm-related infections characterized by high intrinsic resistance (Barman et al., 2019a). Membrane permeabilizers generally operate by interfering with the structural integrity of bacterial membranes. Cationic antimicrobial peptides, such as defensins, cathelicidins, and magainins, act by disrupting bacterial membranes through electrostatic interactions, leading to pore formation and destabilization of lipid bilayers. The synergistic use of these peptides with conventional antibiotics has been highlighted as a promising strategy for enhancing antimicrobial efficacy (Hoque et al., 2016; Hemmati et al., 2021; Kim et al., 2023; Hoque et al., 2015). Synergistic effects of nisin (a polycyclic antibacterial peptide produced by *Lactococcus lactis*), in combination with penicillin and nafcillin against *E. faecalis* and *S. mutans* biofilms, respectively (Tong et al., 2014; Kaletta and Entian, 1989) have been demonstrated. Additionally, small molecules designed to mimic antimicrobial peptides have demonstrated the capacity to destabilize bacterial membranes, leading to increased accumulation of antibiotics within bacterial cells (Ghosh and Haldar, 2015). Novel agents, including cationic biphen [4, 5] arenes (Ghosh and Haldar, 2015; Uppu et al., 2017), have demonstrated both biofilm matrix penetration (uniform diffusion in confocal studies) and broad spectrum bactericidal activity with minimum biofilm inhibitory concentration 50% (MBIC_50_) of 0.62–0.72 μM and minimum biofilm eradication concentrations 50% (MBEC_50_) of 4.94–6.82 μM against both *S. aureus* and *E. coli* biofilms, confirming their ability to not only penetrate the biofilm matrix but also to kill both Gram-positive and Gram-negative bacteria (Du et al., 2023).

#### 4.4.1 Natural and nanotechnological approaches

##### 4.4.1.1 Phytochemical membrane permeabilizers

Naturally occurring plant-derived compounds, including phenolic acids, tannins, terpenoids, alkaloids, essential oils, and quinones can interfere with multiple stages of biofilm development and exert antibiofilm effect (Zhou et al., 2025). Phytochemicals and their metabolites disrupt biofilms through multifaceted actions that converge on membranes, extracellular matrix, and quorum sensing, often with concurrent antibacterial or biofilm dispersal activity (Gonçalves et al., 2023). Membrane targeted mechanisms increase permeability, compromise membrane integrity, and facilitate antimicrobial access to sessile cells, thereby preventing biofilm formation and aiding disruption of established biofilms (Zhou et al., 2025). A study demonstrated that 1,8-cineole (eucalyptol—a naturally occurring compound) exhibited potent antibiofilm activity, achieving a 3-log reduction in viable multidrug-resistant *E. coli* within 1 h of exposure on mature biofilms at sub-MICs and reduced biofilm biomass by 48%−65% across four *E. coli* strains, indicating membrane-permeabilizing action coupled with biofilm disruption (Vazquez et al., 2020). Phytochemicals, such as quercetin and vanillic acid, have also shown promise in enhancing the permeability of the outer membrane, thereby improving the outcome of simultaneous antibiotic treatments. The synergy between these natural compounds and antibiotics can amplify therapeutic efficacy without needing higher treatment doses, consequently reducing potential toxicity (Borges et al., 2016; Vale et al., 2019). The antibacterial activity of phytochemicals cuminaldehyde, indole-3-carbinol, quercetin, and vanillic acid combined with ethylenediaminetetraacetic acid (EDTA), an aminopolycarboxylic acid, was evaluated against *E. coli* and *S. epidermidis* in both planktonic and sessile states, as single- and dual-species cultures. When tested individually, the compounds exhibited only modest antibacterial activity. However, pronounced synergistic effects were observed upon combination with EDTA, particularly with cuminaldehyde and indole-3-carbinol. Near complete (≈100%) biofilm inactivation was achieved in most cases (Vale et al., 2019). Such approaches enable treatment of multi-drug-resistant infections and enhance the overall effectiveness of existing antibiotics against biofilms. While many studies report robust phytochemical antibiofilm activity, the degree of efficacy often depends on compound structure, target organism, delivery context, and biofilm maturity, with some compounds showing limited activity against mature biofilms or requiring synergistic combinations to achieve clinically meaningful disruption (Borges et al., 2016).

##### 4.4.1.2 Nanoparticle technologies

Another innovative approach involves the use of nanoparticle technology as a delivery system for antibiotics combined with permeabilizers (Table 4). Liu *et al*. highlighted the advantages of surface-attaching micellar nanocarriers, improving drug penetration into biofilms by electrostatic targeting of negatively charged bacterial surfaces (Liu et al., 2016). These nanocarriers facilitate sustained and localized release of antibiotics, thereby increasing their bioavailability and therapeutic effectiveness in eradicating biofilm-associated bacteria. For example, pH-responsive mixed-shell polymeric micelles significantly enhanced triclosan delivery throughout *S. aureus* biofilms, resulting in substantially improved biofilm killing compared with the free antimicrobial (Liu et al., 2016). The use of functionalised nanoparticles also takes advantage of their surface properties to enhance interactions with bacterial membranes. For example, surface-engineered gold nanoparticles can disrupt biofilms and display significant bactericidal effects against *S. aureus* and *P. aeruginosa*, which are prominent pathogens in biofilm-associated infections (Giri et al., 2015). Such technological innovations pave the way for targeted treatment modalities that can effectively penetrate and eliminate biofilm structures.

**Table 4:** Nanoparticle technologies, their applications, and mode of action.

Type of the nanoparticle	Mode of action	Specific targets/organism	Effects	References
Micellar nanocarriers	Surface-attaching; improve drug penetration; electrostatic targeting of negatively charged bacterial surfaces; sustained and localized release of antibiotics	Biofilms; negatively charged bacterial surfaces—*S. aureus*	Improve drug penetration, increase antibiotic bioavailability and therapeutic effectiveness	Tong et al., 2014
Surface-engineered gold nanoparticles	Disrupt biofilms; enhance interactions with bacterial membranes	*S. aureus, P. aeruginosa*	Display significant bactericidal effects	Kaletta and Entian, 1989
Nanoparticle-based systems (e.g., liposomes, polymer based-chitosan nanoparticles, metallic nanoparticles including copper nanoparticles (CuNPs), and silver nanoparticles (AgNPs), and Nitric oxide (NO)-releasing silica nanoparticles)	Facilitate direct delivery of antibacterial agents into biofilms; improved penetration and retention, promote direct membrane and matrix disruption, and efflux inhibition	Biofilms; bacterial membranes, efflux pump, and biofilm matrix (*P. aeruginosa, E. coli, S. aureus, S. epidermidis*, and *C. albicans)*	Enhance efficacy of antibacterial agents in biofilm environments	Pattnaik et al., 2024; Kvist et al., 2008; Guo et al., 2021; Vale et al., 2019

#### 4.4.2 Synthetic and hybrid membrane disruptors

##### 4.4.2.1 Synthetic polymers and macromolecules

The development of cationic macromolecules has been greatly effective at targeting bacterial membranes and overcoming antibiotic resistance. Uppu et al. (2016) demonstrated that cationic and amphiphilic polymers could disrupt established biofilms and enhance the action of conventional antibiotics against pathogen such as *A. baumannii* (83%−84% biofilm biomass reduction). Newly synthesized guanidinium-functionalised compounds such as GP5 have demonstrated excellent antibacterial potency against both *E. coli* and *S. aureus* (MIC values of 12.5 μM and 6.3 μM, respectively) (Guo et al., 2021). Amino-acid conjugated polymers have also emerged as viable candidates in this space. Studies indicate that these polymers can enhance the antibacterial activity of co-administered antibiotics such as rifampicin against resistant Gram-negative isolates (Barman et al., 2019b). The versatility in designing polymeric structures allows for tailored approaches to combat increasing rates of resistance.

#### 4.4.3 Naturally occurring peptides: benefits and limitations

Alongside these synthetic polymers and nanocarrier systems, naturally occurring peptides such as polymyxins remain the best-known membrane permeabilizers, though due to their toxicity they are limited in use. Polymyxins belong to the group of non-ribosomal peptides (Trimble et al., 2016). They are naturally occurring and produced by Gram-positive bacterial species such as *Paenibacillus polymyxa* (Raza et al., 2008) and therefore, only active against Gram-negative strains. Despite their high potency, they are generally reserved as last resort antimicrobials, as their non-selective action on outer membranes causes extensive side effects, which include kidney and liver damage. An increased effort into the development of active derivatives with reduced toxicity has seen a rise in research as stand-alone as well as in combination therapy (Meade et al., 2020). However, the increasing use of polymyxins and the rise in plasmid-borne colistin resistance through modification of lipid A and subsequently reduction in polymyxin affinity could bring down the last line of defense and be the beginning of truly pan resistant infections (Trimble et al., 2016).

#### 4.4.4 Emerging strategies: lipopeptides, small molecules, and hybrid systems

Recent studies have increasingly focused on diverse classes of membrane active compounds to combat the growing challenge of antibiotic-resistant bacteria, particularly when addressing biofilms. Lipopeptides, particularly daptomycin, surfactin, and iturins, exhibit similar mechanisms by integrating into bacterial membranes and inducing structural perturbations. These compounds predominantly target Gram-positive bacteria, showcasing significant antibacterial activity that is comparable to known antibiotics like colistin used mainly against Gram-negative bacteria (Paduszynska et al., 2019). The combination of lipopeptides and other antibiotics, such as vancomycin, has demonstrated improved outcomes against multidrug-resistant strains, further establishing their value in combination therapies (Claeys et al., 2014).

Small molecule membrane disruptors, such as arylamide oligomers and ceragenins, have emerged as novel options to depolarize membranes and increase permeability, which are crucial for tackling resistant bacterial strains. Their potential application as adjuvants plays a critical role in overcoming the defensive barriers posed by biofilms (Kim et al., 2023). Additionally, dendrimers, which are multivalent macromolecules with cationic surfaces, exhibit the capacity to disrupt bacterial envelopes through multivalent interactions, representing yet another innovative strategy in the fight against bacterial pathogens (Werner et al., 2024).

In the context of hybrid or dual-function compounds, peptide-antibiotic conjugates show promise by combining membrane permeabilization with targeted drug delivery mechanisms, thereby enhancing efficacy in biofilm environments (Arias et al., 2010). Nanoparticle-based systems, such as liposomes and metallic nanoparticles, also facilitate the direct delivery of antibacterial agents into biofilms while promoting the disruption of the membrane integrity (Schneider-Futschik et al., 2018). Collectively, these strategies reflect the breadth of ongoing research aimed at developing effective treatments against biofilm associated infections.

### 4.5 Metabolome activators as antibiotic enhancers in biofilms

Building on strategies targeting bacterial envelopes, metabolome activators offer a diverse approach in targeting the intrinsic metabolic resistance of biofilm associated bacteria. These compounds can reshape bacterial metabolism, effectively reactivating persister-like and slow growing subpopulations that would otherwise exhibit reduced susceptibility to antibiotics (Su et al., 2015; Schrader et al., 2021). By reprogramming bacterial metabolic pathways, these compounds restore a physiological state conducive to antibiotic action, thereby restoring antibacterial efficacy.

The success of many antibiotics, including aminoglycosides and β-lactams, depends on active bacterial biosynthetic pathways, which require active processes such as respiration and the generation of reactive oxygen species (ROS) (Wang et al., 2018; Zou et al., 2018). Bacteria in biofilms experience steep gradients of oxygen, nutrients, and metabolic waste across the extracellular matrix, leading to heterogeneous metabolic states. Therefore, a substantial proportion of embedded cells adopt a dormant or slow growing state, displaying reduced susceptibility to antibiotics. Metabolomic activators counteract this tolerance by stimulating key metabolic pathways, including the tricarboxylic acid (TCA) cycle and the electron transport chain (ETC), resulting in restored energy generation and ROS production, and ultimately reinstating antibacterial susceptibility (Wang et al., 2020; Yan et al., 2023). Recent work in AMR research identified two approaches in nutrient-based enhancer strategies: metabolite-driven and metabolic-state-driven (Peng et al., 2025). The metabolite-driven method relies on empirical observations to link specific nutrient supplementation to increased bacterial efficacy of known antibiotics. In contrast, the metabolic-state-driven approach identifies differences in metabolic profiles between resistant and susceptible bacteria, aiming to correct the imbalance and reinstate antibacterial vulnerability.

#### 4.5.1 Carbon source supplementation

Carbon substrates provide energy and act as electron donors, fuelling respiration and producing the proton motive force (PMF), which provides the means for active antibiotic uptake into the cell. Carbon substrates including glucose, mannitol, fructose, and pyruvate have been shown to potentiate aminoglycoside antibiotics by three orders of magnitude, killing *E. coli* and *S. aureus* persister cells by generating a proton motive force that enhances aminoglycoside uptake, effectively eradicating persisters and improving treatment outcomes in a mouse infection model (Allison et al., 2011). Fructose has been shown to reinstate aminoglycoside susceptibility in multidrug-resistant *Edwardsiella tarda, K. pneumoniae, and* MRSA by enhancing flux through the ETC and elevating ATP levels (*E. tarda* cell viability reduced by 2.5–155.9-fold with varying doses of fructose in combination with 500 μg/mL kanamycin) (Su et al., 2015). Supplementation with fumarate or glucose has been shown to improve uptake of antibiotics like gentamicin by restoring respiratory activity (Zhang et al., 2020; Donkor et al., 2023). The effect of carbon source activation extends to antibiotics that target protein synthesis, as the additional energy fuels metabolic processes, including cell division and protein synthesis. For example, mannitol (10–40 mM) restored aminoglycoside susceptibility in *P. aeruginosa* biofilms, increasing the sensitivity of persister cells to tobramycin by up to 1,000-fold) (Barraud et al., 2013)

#### 4.5.2 Stimulation of the TCA cycle

The stimulation of the TCA cycle is another beneficial strategy to enhance antibiotic action. Activating this cycle boosts oxidative metabolism processes, ultimately leading to increases in ATP and ROS production. In *S. aureus*, the inactivation of the TCA cycle via deletion of *sucA* or *fumC* genes results in a decrease in intracellular ATP and a reduction in ROS production, correlating to a 100- to 1,000-fold increase in persisiter cell formation. These metabolic changes are crucial for the bactericidal activity of antibiotics like β-lactams and aminoglycosides (Wang et al., 2018; Zou et al., 2018). Supplementation with carbon sources such as fructose, glucose, or mannitol reduces persister cell formation in *E. coli* and *S. aureus* by up to 1,000-fold. Additionally, supplementation with pyruvate has been shown to improve the effectiveness of gentamicin and ampicillin, while other metabolic intermediates such as succinate and malate have been associated with increased sensitivity of bacterial persister cells in Gram-negative bacteria to a range of antibiotics (Schrader et al., 2021; Yan et al., 2023; Allison et al., 2011; Barraud et al., 2013). However, the effect of these metabolites can be species-specific, as in *E. tarda*, the addition of succinate, fumarate, or malate has been shown to increase viability against chloramphenicol by 3.83 to 153.9-fold (Wang et al., 2020).

#### 4.5.3 Amino acid mediated metabolic activation

Amino acid metabolism also plays a vital role in enhancing antibiotic efficacy through metabolic stimulation. Specific amino acids can increase redox turnover and support respiratory activity by serving as substrates or cofactors in metabolic pathways. Arginine has been reported to improve the effectiveness of gentamicin against *P. aeruginosa* biofilms (Schrader et al., 2021; Ye et al., 2018). Similarly, glutamate has been found to enhance the efficacy of ciprofloxacin by increasing the production of ROS production (Basu et al., 2018). Zhao et al. showed that L-glutamine enhances uptake of antibiotics such as aminoglycosides, β-lactams, quinolones, and tetracyclines, potentiating their killing of uropathogenic *E. coli*, as well as *P. aeruginosa, K. pneumoniae*, and *A. baumannii*. In uropathogenic *E. coli*, the addition of L-glutamine increased antibiotic uptake by 2–3-fold and decreased bacterial survival by 10,000-fold. This reversal occurs through a pathway that modulates membrane permeability via OmpF, an integral outer membrane protein that acts as a passive pore allowing influx of small hydrophilic molecules into the cell (Zhao et al., 2021).

#### 4.5.4 ROS inducing agents

Ethylene agents are small molecules that induce the production of ROS within the bacterial cell. ROS are highly chemically reactive and readily damage intracellular components such as DNA, proteins, and lipids, a process defined as oxidative stress. Compounds such as menadione and plumbagin increase intracellular levels of ROS and have been linked to improved efficacy of ciprofloxacin and gentamicin (Van Acker et al., 2016). In *Burkholderia cepacia*, the combination of 100 μM menadione with ciprofloxacin resulted in a 2–3-log reduction in CFU/mL, significantly improving antibacterial efficacy (Van Acker et al., 2016).

#### 4.5.5 Modulation of secondary messenger systems

In addition to supplementing primary metabolic substrates, targeting secondary messenger systems also constitutes an innovative approach to enhance antibiotic efficacy. The inhibition of the stringent response, which involves the signal molecule (p)ppGpp (hyperphosphorylated guanosine derivatives), can restore antibiotic susceptibility by preventing metabolic downshift during times of environmental stress (Meade et al., 2020; Rekha et al., 2020; Gaca et al., 2015). Furthermore, analogs of cAMP have demonstrated positive effects in increasing β-lactam activity (Schrader et al., 2021). Host-derived metabolites, such as bicarbonate and lactate, have been shown to mimic conditions *in vivo* that activate metabolic processes. For example, physiological concentrations of bicarbonate (25–44 mM) have been shown to lower the MIC of β-lactam antibiotics (cefazolin and oxacillin) from 16 μg/mL to 4 μg/mL at 25 mM NaHCO3 and further to 0.5–1 μg/mL at 44 mM NaHCO_3_, representing multiple-fold reductions in resistance in bicarbonate-responsive strains (Ersoy et al., 2019; Rekha et al., 2020).

### 4.6 Implications for antimicrobial therapy

The use of metabolome activators alongside conventional antibiotics indicates a shift in antimicrobial therapy. Both metabolome-based strategies, one relying on nutrient supplementation and the other on metabolic-state-driven differences (Table 5), offer ways to sensitize biofilm embedded and persister-like populations (Schrader et al., 2021; Zou et al., 2018). Ongoing research into how specific metabolites, metabolic pathways and antibiotics interact will be key to improving treatment outcomes.

**Table 5:** Metabolome activators, metabolic state-driven, and metabolite-driven distinction.

Metabolome activator	Category	Mechanism/ target	Antibiotic/ effect	Organism	References
Carbon substrates					
Mannitol	Metabolite-driven	Provides energy and electron donors; enhances ETC flux and ATP	Enhances tobramycin efficacy; disrupts biofilms	*P. aeruginosa*	Barraud et al., 2013
Fumarate	Metabolite-driven	Restores respiratory activity; supports PMF	Improves gentamicin uptake	*E. coli*	Zhang et al., 2020
TCA cycle intermediates					
Pyruvate	Metabolic-state-driven	Stimulates TCA cycle; increases ATP and ROS	Improves gentamicin and ampicillin efficacy	Gram-negative persisters	Zhang et al., 2020; Donkor et al., 2023
Succinate	Metabolic-state-driven	Boosts TCA cycle; increases ROS	Increases persister susceptibility	Gram-negative	Schrader et al., 2021; Zhang et al., 2020
Malate	Metabolic-state-driven	TCA cycle intermediate; enhances oxidative metabolism	Increases persister susceptibility	Gram-negative	Schrader et al., 2021; Zhang et al., 2020
Amino acids					
Arginine	Metabolic-state-driven	Enhances redox turnover; supports respiration	Enhances gentamicin activity	*P. aeruginosa* biofilms	Schrader et al., 2021
L-Glutamine	Metabolite-driven	Feeds into purine metabolism, increases antibiotic uptake	Enhances aminoglycosides, β-lactams, quinolones, and tetracyclines	*E. coli, P. aeruginosa, K. pneumoniae, A. baumannii*	Zhao et al., 2021
Glutamate	Metabolic-state-driven	Increases ROS production	Synergizes with ciprofloxacin	Gram-negative	Warraich et al., 2020
ROS-inducing agents					
Menadione	Metabolite-driven	ROS-inducing agent	Improves ciprofloxacin efficacy	Gram-negative	Rekha et al., 2020; Kohanski et al., 2007
Plumbagin	Metabolite-driven	ROS-inducing agent	Improves gentamicin efficacy	Gram-negative	Rekha et al., 2020; Kohanski et al., 2007
Secondary messenger/host metabolites					
(p)ppGpp inhibitors	Metabolic-state-driven	Inhibit stringent response; prevent metabolic downshift	Restore antibiotic susceptibility under stress	MRSA, *P. aeruginosa*	Hauryliuk et al., 2015
cAMP analogs	Metabolic-state-driven	Modulate secondary messenger; enhance metabolism	Increases β-lactam activity	Gram-negative	Schrader et al., 2021
Bicarbonate	Metabolic-state-driven	Mimic *in vivo* metabolic activation	Sensitize to standard antibiotics	MRSA, *P. aeruginosa*	Ersoy et al., 2019, 2017

### 4.7 Quorum sensing inhibitors (QSIs)

Quorum sensing, a system of communication for bacterial cells, is crucial during biofilm formation. Quorum sensing inhibitors (QSIs) play a significant role in enhancing the efficacy of antibiotic treatments and have now come into view as promising agents in combination therapies to combat antibiotic resistance, specifically in biofilm-associated infections. A combination of cinnamaldehyde (CAD) and colistin (COL) demonstrated synergistic activity against *P. aeruginosa*, remarkably inhibiting biofilm formation and dispersing preformed biofilms (75.2% inhibition, and 90% dispersion) (Topa et al., 2020). CAD also exhibited additive activity with tobramycin (TOB) in biofilm inhibition and dispersion in GFP reporter assays (83.9% and 90%, respectively) (Topa et al., 2020). This synergy suggests that QSIs may serve as valuable adjuncts in treating biofilm-associated infections. Albendazole, a clinical drug belonging to an anthelmintic class as niclosamide (an FDA-approved clinical drug recently found to exhibit QSI activity) showed QSI activity and, when combined with TOB, efficaciously inhibited biofilm formation in *P. aeruginosa* (Singh and Bhatia, 2018). QSIs signaling disruptors derived from drugs such as aspirin, and piroxicam have also demonstrated ability to inhibit biofilm formation in bacteria such as *P. aeruginosa* (Dsouza et al., 2024). For instance, aspirin at 6 mg/mL significantly attenuated quorum sensing activity in *P. aeruginosa* (*p* < 0.01), accompanied by marked reductions in motility and biofilm formation (*p* < 0.01). This effect was associated with downregulation of key quorum sensing genes, with expression of *lasI, lasR, rhlI, rhlR, pqsA*, and *pqsR* reduced by 38%, 72%, 69%, 72%, 74%, and 43%, respectively (El-Mowafy et al., 2014).

The exploration of natural QSIs from plants, microorganisms, and marine organisms offers promising pathways for the development of QSIs (Alum et al., 2025). Plant derived QSIs such as ajoene extracted from garlic (*Allium sativum*), curcumin from turmeric (*Curcuma longa*), epigallocatechin gallate (EGCG) compound extracted from tea (*Camellia sinensis*), flavonoids (hesperidin, naringenin, kaempferol) from citrus fruits, piperine from pepper (*Piper nigrum*), cinnamaldehyde from cinnamon (*Cinnamomum verum*), proanthocyanidins, from cranberry (*Vaccinium macrocarpon*), and resveratrol from grapes (*Vitis vinifera*) among others have demonstrated quorum sensing inhibition mechanisms in bacteria such as *P. aeruginosa, E. coli, Vibrio spp*., and *Salmonella* (Alum et al., 2025; Brackman et al., 2011). Similarly, QSI compounds obtained from marine organisms, such as indole alkaloids from coral-associated bacteria, lactones, alkaloids from marine bacteria, xanthones, terpenoids, from marine fungi, furanones from marine algae, and *N*-acylhomoserine lactone (AHL) analogs from marine Actinomycetes have shown potent QS-inhibitory activity (Alum et al., 2025). Microbial derived QSIs such as surfactin produced by *Bacillus spp*., rhamnolipid from *Pseudomonas spp*., furanones from *Aspergillus spp*., cyclic dipeptides from *Lactobacillus spp*., AHL lactonase from *Variovorax paradoxus*, and autoinducer-2 (AI-2) inhibitors from *Bacillus cereus* have also shown QS inhibitory effects (Alum et al., 2025). Phyto chemicals moracin M extracted from *Morus alba* L. (Moraceae), and cannabigerolic acid obtained from cannabigerol- and cannabidiol-rich cultivars of *Cannabis sativa* demonstrated significant inhibition of AI-2 production in MRSA cultures and *V. harveyi* (Helcman et al., 2025). This suggests their potential for further development as antibiofilm agents targeting quorum-sensing inhibition.

Additionally, nanoparticles have emerged as promising quorum sensing inhibitors because of their ability to interact with bacterial cells and signaling molecules such as acyl-homoserine lactones or AI-2. In a past study, biosynthesized silver nanoparticles (AgNPs) at sub-MIC (50 μg/mL) reduced the expression of QS-regulated genes including *algC, pslA*, and *pelA* by 77%, 83%, and 68%, respectively, and suppressed *P. aeruginosa* biofilm formation by 78%, indicating their potential as antibiofilm agents (Aflakian and Hashemitabar, 2025). NPs have a potential application despite concerns regarding toxicity (Qayyum and Khan, 2016).

#### 4.7.1 Challenges and prospects

Several QSIs developed in the past have failed to reach clinical application due to known toxicity effects, challenges related to specificity, pharmacokinetic limitations, bacterial adaptability, and limited effectiveness leading to resistance (Singh and Bhatia, 2018). For example, despite biaromatic furanones and brominated pyrrolones demonstrating QSI activity, many of these compounds have exhibited potent cell toxicity, limiting their clinical application (Liu et al., 2020). Furthermore, while CAD combined with COL showed synergistic activity, CAD combined with other antibiotics such as carbenicillin, TOB, or erythromycin did not show synergistic activity in checkerboard assays, signifying their limited efficacy (Topa et al., 2020). Continued research focused on multi-target QS inhibitors, rigorous preclinical assessment, and advanced delivery systems, to overcome current limitations is crucial to harness their full potential and address the challenges associated with their clinical application (Touati et al., 2025).

## 5 Conclusion and future perspectives

The exploration of resistance breakers opens new avenues to extend the clinical applicability of existing antimicrobials, addressing the challenges posed by antibiotic resistance and biofilm associated infections. The future of antibiotic resistance breaker development may lie in combination therapies, where resistance breakers are used alongside antibiotics to enhance their clinical efficacy against resistant strains. As demonstrated in various studies, resistance breakers show potential in enhancing clinical efficacy of antibiotics, reducing virulence and altering bacterial community dynamics. For example, β-lactamase inhibitors have shown viable clinical potential, and efflux pump inhibitors, membrane targeting compounds, and compounds targeting host processes (immunomodulatory peptides) may be a solution to biofilm and non-biofilm associated infections such as sepsis. The metabolic state-driven nutrient-based approach, a strategy focused on utilizing nutrient metabolites as metabolic reprogramming agents to restore lethal effect of antibiotics by promoting antibiotic uptake in bacteria, has emerged. Glutamine and pyruvate have been shown to promote antibiotic (aminoglycoside, β-lactam) uptake in resistant bacteria (Peng et al., 2025; Zhao et al., 2021).

Currently, there is a lack of *in vitro* biofilm models that accurately mimic the host environment. This gap explains why most resistance breakers that work *in vitro* poorly translate into clinical efficacy. To improve this outcome, development of biofilm models that accurately reflect the physiological complexity in biofilm development is required, including models that reflect the microenvironment and chemical heterogeneity in the host (gradients of oxygen, and metabolites), capture host-pathogen interactions (such as nutrient availability and immune responses), and allowing testing under clinically realistic conditions (such as the presence of host proteins, and polymicrobial infections). Employing complex biofilm models such as flow cell and microfluidic models may provide continuous nutrient flow, and shear stress, mimicking environments like catheters. Similarly, incorporating human- or animal-derived tissue can provide a realistic substrate, for example the *ex vivo* pig lung model to study biofilms in cystic fibrosis, reproducing oxygen and nutrient gradients. Organotypic and 3D cell culture models involving the use of organoids, or human epithelial cells may also be used to study host-biofilm interactions.

Translating resistance breakers from bench to clinical setting requires thorough demonstration of synergistic efficacy with co-therapy antibiotics, target engagement, favorable pharmacokinetics/pharmacodynamics (PK/PD), and acceptable safety in humans. Additionally, overcoming the limitations of existing resistance breakers will require application of innovative delivery strategies that will enhance localization, bioavailability, and stability at infection sites. Phage-mediated systems and nanotechnology-based carriers show promise in this area. Nanoparticles play an important role in target delivery of antimicrobial agents to the required sites, and a wide range of nanoparticles including silver, zinc, copper, oxide, and quantum dots have shown significant potential in inhibiting biofilms owing to their antibacterial properties (Dsouza et al., 2024; Pereira et al., 2024). Nanomaterials can be engineered to co-deliver resistance breakers and antibiotics in a controlled and targeted manner, while bacteriophages together with their enzymes offer the advantages of biofilm penetration and bacterial specificity.

Although resistance breakers are a promising strategy, it is important to note that bacteria simultaneously utilize diverse resistance mechanisms, therefore making it challenging to develop antibiotic resistance breakers that can counteract all of them. Thus, continued research and development in this field is needed to leverage the full potential of resistance breakers and ultimately combat the growing threat of resistant bacterial infections.
